# Natural Compounds Targeting Cancer-Associated Fibroblasts against Digestive System Tumor Progression: Therapeutic Insights

**DOI:** 10.3390/biomedicines10030713

**Published:** 2022-03-19

**Authors:** Kuan-Jung Chiu, Hsin-Ying Clair Chiou, Chi-Han Huang, Pin-Chun Lu, Hui-Ru Kuo, Jiunn-Wei Wang, Ming-Hong Lin

**Affiliations:** 1 Department of Microbiology and Immunology, School of Medicine, College of Medicine, Kaohsiung Medical University, Kaohsiung 807, Taiwan; u107001084@kmu.edu.tw; 2 Center of Teaching and Research, Kaohsiung Municipal Siaogang Hospital, Kaohsiung Medical University, Kaohsiung 812, Taiwan; phoenixchiou@gmail.com; 3 Kaohsiung Medical University Hospital, Kaohsiung Medical University, Kaohsiung 807, Taiwan; 4 Graduate Institute of Medicine, College of Medicine, Kaohsiung Medical University, Kaohsiung 807, Taiwan; cheryl60286@gmail.com (C.-H.H.); zsp93237@gmail.com (P.-C.L.); a0976566730@gmail.com (H.-R.K.); 5 Department of Internal Medicine, School of Medicine, College of Medicine, Kaohsiung Medical University, Kaohsiung 807, Taiwan; 6 Department of Gastroenterology, Division of Internal Medicine, Kaohsiung Medical University Hospital, Kaohsiung Medical University, Kaohsiung 807, Taiwan; 7 Graduate Institute of Clinical Medicine, College of Medicine, Kaohsiung Medical University, Kaohsiung 807, Taiwan; 8 Department of Medical Research, Kaohsiung Medical University Hospital, Kaohsiung Medical University, Kaohsiung 807, Taiwan; 9 M.Sc. Program in Tropical Medicine, College of Medicine, Kaohsiung Medical University, Kaohsiung 807, Taiwan

**Keywords:** tumor microenvironment, cancer-associated fibroblasts (CAFs), digestive system cancers, gastrointestinal cancer, natural products

## Abstract

Cancer-associated fibroblasts (CAFs) are critical for cancer occurrence and progression in the tumor microenvironment (TME), due to their versatile roles in extracellular matrix remodeling, tumor–stroma crosstalk, immunomodulation, and angiogenesis. CAFs are the most abundant stromal component in the TME and undergo epigenetic modification and abnormal signaling cascade activation, such as transforming growth factor-β (TGF-β) and Wnt pathways that maintain the distinct phenotype of CAFs, which differs from normal fibroblasts. CAFs have been considered therapeutic targets due to their putative oncogenic functions. Current digestive system cancer treatment strategies often result in lower survival outcomes and fail to prevent cancer progression; therefore, comprehensive characterization of the tumor-promoting and -restraining CAF activities might facilitate the design of new therapeutic approaches. In this review, we summarize the enormous literature on natural compounds that mediate the crosstalk of CAFs with digestive system cancer cells, discuss how the biology and the multifaceted functions of CAFs contribute to cancer progression, and finally, pave the way for CAF-related antitumor therapies.

## 1. Introduction

**Figure 1:**
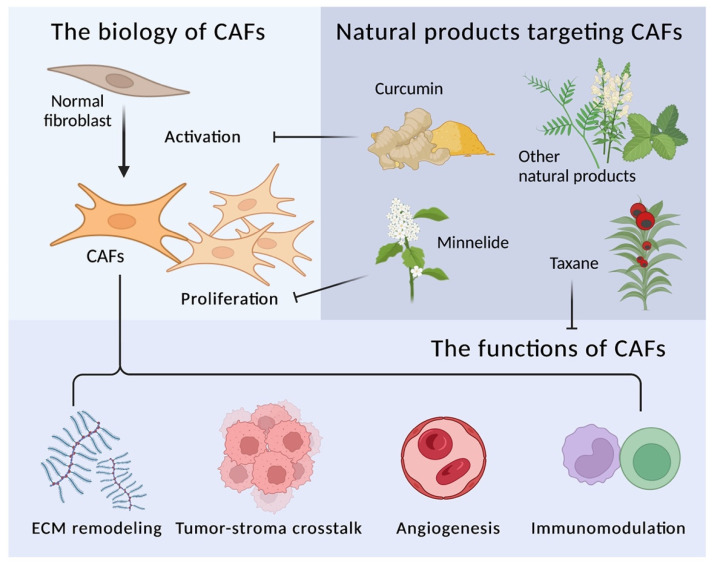
The therapeutic insights of natural compounds targeting CAFs (cancer-associated fibroblasts). CAFs in the TME (tumor microenvironment) are the critical contributor of tumorigenesis, cancer progression, and metastasis. The therapeutic strategies targeting CAFs are still flourishing. Several natural products have been discovered to have the potential to inhibit CAFs activation, proliferation, and tumor-promoting functions.

The tumor microenvironment (TME), also known as tumor stroma, is a heterogenous component comprising fibroblasts, immune cells, endothelial cells, and noncellular constituents including blood vessels, the extracellular matrix (ECM), and the basement membrane, as well as various cytokines, chemokines, and growth factors [1,2]. The concept of TME can be traced back to the “seed and soil” theory, which describes the reciprocal relationship of tumor (seed) and TME (soil). The critical role of TME in promoting cancer initiation, progression, and recurrence has received widespread attention in recent years [3,4,5]. Cancer-associated fibroblasts (CAFs) are the most abundant stromal component in TME [4,6,7,8], and promote cancer progression through the sophisticated interaction of their four main components in the TME, i.e., ECM, cancer cells, endothelial cells, and immune cells [2,7]. Versatile therapeutic strategies targeting CAF are now widely used [6]. Other than traditional therapies, some of the distinct natural compounds have also been identified as CAF targets by the modulation of specific signaling pathways, kinases, enzymes, or epigenetic changes. The modulations consequently influence interesting tumor–stroma crosstalk in the TME and are related to patient prognosis, which indicates that natural products might have the exciting potential for novel cancer therapy development and are worth studying [9,10]. The general concept of the current review was diagramed in Figure 1.

## 2. The Biology and Functions of CAFs

### 2.1. Biology of CAFs

The assumption that “tumors are wounds that do not heal” introduced by Harold F. Dvorak first pointed out that tumor shares a similarity with the wound-healing process, which created a research niche exploring the relationship between tumors and fibroblasts [11].

During the wound-healing process, myofibroblasts, i.e., activated normal fibroblasts, play a central role in the ECM remodeling and wound contraction process [12]. In the TME, the protagonist is certainly CAFs derived from multiple origins, including local fibroblasts, bone marrow-derived stem mesenchymal stem cells, epithelial cells, endothelial cells, even pericytes, and adipocytes. These cells are recruited and activated by cancer parenchyma and then experience a conversion into activated CAFs, which display distinct properties that are different from local fibroblasts [2,4,6]. Other than common genetic alterations in the genome of cancer cells, the phenotype of CAFs is maintained mainly by abnormal signaling cascade activation and epigenetic modification [13]. One of the key factors regulating the conversion of normal fibroblasts to CAFs is transforming growth factor-β (TGF-β). The main isoform TGF-β1 activates the Smad-dependent [14,15] as well as Smad-independent pathway in CAFs to induce CAF markers’ expression and multiple pro-malignant properties, such as the contractility and cytokine secretion functions [16,17]. Therefore, TGF-β becomes a potential therapeutic target for cancer therapy [18,19]. For another example, the Wnt signaling pathway has played a critical role in intestinal cancer and tumor immunity [20,21], and also induces the switching of CAFs’ phenotypes and promotes tumor growth and progression [22]. Different epigenetic alterations shape the distinct characteristic of CAFs. Posttranscriptional modification (PTM) regulated by micro-RNA (miRNA) has been studied in the TME of several cancers [23]. The expression level of miRNA is related to the pro-metastasis functions and several signaling pathways in CAFs [24]. In addition, aberrant DNA methylation, especially in the promoter region, also influences the epigenetic changes of CAFs in tumorigenesis [23]. Overall, multiple complicated mechanisms are involved in the conversion and activation of CAFs. In the next section, we describe several promising natural products, which have the potential to target these related pathways, and might lead to a novel cancer treatment strategy in the future.

CAFs are a highly heterogeneous population, and the classification of CAFs is according to their versatile origins, phenotypes, and biological functions and supports more accurate and patient-specific CAFs-targeting therapies [25]. Therefore, identification of CAFs markers is necessary. For example, commonly used activated CAF markers include the fibroblast activation protein (FAP), α-smooth muscle actin (α-SMA), collagen I, and platelet-derived growth factor receptor α/β (PDGFRα/β). Some of them also serve as prognostic markers and become therapeutic targets [26]. However, the high heterogeneity of CAFs also poses challenges in the anti-CAFs therapies’ development [27].

The metabolism of CAFs shows a distinct characteristic. Most tumor cells tend to use aerobic glycolysis rather than oxidative phosphorylation to generate energy and metabolites, which is called the “Warburg effect”. However, recent studies showed that this model is not applied in all types of cancer but is related to the original property and the tumor-surrounding stroma. Therefore, a novel model of the “reverse Warburg effect” has been established [28]. The reverse Warburg effect describes the metabolic symbiosis between tumor and stromal cells where CAFs undergo a metabolic switch to aerobic glycolysis to provide cancer cells with sufficient metabolic products and nutrients. CAFs are the critical cell type that contributes to this distinct metabolic reprogramming [29], which is characterized by the upregulation of glycolytic enzymes, such as hexokinase and phosphofructokinase, and also the enhancement of metabolite transporters such as monocarboxylate transporter 1 and glucose transporter 1, and the loss of caveolin-1 (Cav-1). The process might have an association with the hypoxia-inducible factor-1α, and TGF-β signaling and is often considered a hallmark of cancer progression. Therefore, drugs targeting the specific signaling pathway or the related enzymes might potentially pose an advantage to cancer therapy [30].

### 2.2. Functions of CAFs

#### 2.2.1. ECM Remodeling

**Figure 2:**
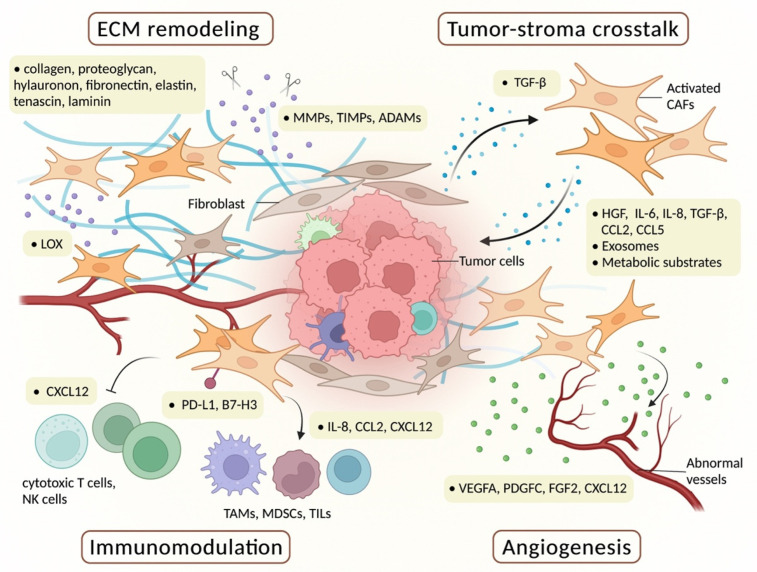
The multifaceted functions of CAFs (cancer-associated fibroblasts) in the TME (tumor microenvironment). Cancer cells can activate tumor-surrounding fibroblasts and other cells by TGF-β and other signaling pathways to become CAFs, which modulate the TME and promote cancer progression by four aspects: (1) CAFs controlling the abnormal and pro-metastasis ECM (extracellular matrix) remodeling by producing and crosslinking ECM components, and the ECM might be degraded by MMPs (matrix metalloproteases) and other enzymes. (2) CAFs interact with cancer parenchyma through multiple kinds of growth factors, cytokines, and chemokines, such as HGF, IL-6, IL-8, CCL2, and CCL5. CAFs also secret metabolic substrates to support tumor metabolism. CAFs also produce exosomes mainly containing miRNA to regulate tumor cells. (3) CAFs promote the abnormal angiogenesis of cancer by producing VEGFA, PDGFC, FGF-2, and CXCL12. (4) CAFs exert a modulatory role of the tumor-infiltrating immune cells, such as TAMs, MDSCs, TILs, cytotoxic T cells, and NK cells.

The ECM is composed of collagens, proteoglycans, glycosaminoglycans (such as hyaluronan), and glycoproteins (such as fibronectin, elastin, tenascins, and laminins) (Figure 2). Dysregulation of the ECM homeostasis is the hallmark of cancer progression [31,32]. CAFs play a pivotal role in the pro-tumorigenic ECM remodeling by ECM synthesis, crosslinking, degradation, and signaling transduction [33].

ECM stiffness is a characteristic of tumorigenesis and supports the malignant properties of cancer cells. The role of CAFs is to produce and crosslink ECM components such as collagen fibers and hyaluronan [34]. CAFs-derived lysyl oxidase (LOX) functions as the initiator of collagen crosslinking in overexpression in several cancers, such as gastric and breast cancer and promoting cancer cell survival, epithelial–mesenchymal transition (EMT), metastasis, invasion, angiogenesis, and drug resistance [35]. In addition, CAFs modulate TME during invasion and angiogenesis by dysregulating the production of ECM-degrading enzymes such as matrix metalloproteases (MMPs) and tissue inhibitors of metalloproteinase (TIMPs). Among them, MMP-2 and MMP-9 are well-studied and are closely related to cancer progression [36]. CAFs also increase the expression of disintegrin and metalloproteinases (ADAMs), which belong to the superfamily of MMPs, and modulate tumor progression [37]. In an enzyme-independent manner, CAFs also use physical contractility to pull the basement membrane and promote cancer invasion [38]. CAFs modulate fibronectin deposition by integrin α5β1 in pancreatic ductal carcinoma [39], and by integrin αvβ3 in colon cancer [40], thus promoting cancer cell directional migration. Taken together, ECM remodeling is one of the pivotal effects of CAFs’ functions, and thus, at least, serves as a therapeutic target for antitumor treatment.

#### 2.2.2. Tumor–Stroma Crosstalk

Tumor–stroma crosstalk is the most predominant tumor-promoting role of CAFs via direct contact and indirect paracrine secretion (Figure 2). The mechanical interaction between N-cadherin on the CAFs’ membrane and E-Cadherin on cancer cells consequently enhances cancer metastasis [41]. However, the most well-studied function of CAFs is secretome production, which indirectly and profoundly modulates TME and cancer progression. Various CAF-derived soluble factors including growth factors, cytokines, and chemokines play a critical role in stimulating tumor growth, metastasis, EMT, angiogenesis, and chemotherapy resistance [1,2,4,42].

Hepatocyte growth factor (HGF) is mainly secreted by CAFs and binds to the receptor, MET, on cancer cells, which activates the downstream signaling involving AKT, ERK/MAPK, and STAT3, and then enhances cancer cell survival, EMT, migration, invasion, proliferation, and chemotherapy resistance [43]. Interleukin-6 (IL-6) regulates multiple pro-malignant functions of CAFs in digestive system cancers by binding the IL-6R and activating the JAK kinase and the downstream pathway. For instance, CAF-derived IL-6 supports cancer cells’ EMT and metastasis by IL-6/STAT signaling in colorectal cancer [44], gastric cancer [45], and pancreatic cancer [46]. IL-6 also enhances the chemoresistance via the STAT3 pathway in esophageal [47,48,49,50] or gastric cancer [51] and via the JAK2/BECN1 pathways in colorectal cancer [52]. Similarly, interleukin-8 (IL-8) is demonstrated to modulate doxycycline [53] and cisplatin [54] resistance via the nuclear factor-kappa B (NF-κB) signaling pathway.

TGF-β signaling is commonly upregulated in the TME. Cancer cells secrete TGF-β and induce transformation of normal fibroblasts to CAFs. CAFs also autocrine TGF-β to regulate the pro-malignancy phenotype, and reciprocally contribute to cancer progression [55]. CAF-derived C-C motif chemokine ligand 2 (CCL2) and CCL5 activate the Hedgehog (Hh) signaling pathway, and CCL7 and CXCL16 are involved in the TGF-β pathway [56]. In addition, some types of ECM components also exert multifaceted functions of signaling transduction. Osteopontin (OPN) is one of the examples that upregulates and promotes cancer cell proliferation, migration, and invasion, and is correlated with poor prognosis [57].

The communication of CAFs and tumor cells are partly mediated by exosomes [58]. Exosomes are small vesicles delivering RNA, mRNA, micro-RNA (miRNA), circular RNA (circRNA), long noncoding RNA (lncRNA), DNA, and proteins [2,59]. Dysregulated miRNAs are the main exosomal components derived from CAFs and not only modulate the biological characteristics of CAFs, but are also a multifaceted modulator of the crosstalk between cancer cells and stromal cells. Exosomal miRNA often targets distinct pathways such as CCL2, IL-6, IL-8, WNT, and PI3K and remodels the pro-malignant properties of tumor cells [60]. In gastric cancer, miRNA-149 mediates cancer cell EMT, invasion, and stemness through prostaglandin E2/IL-6 signaling in CAFs [61]. On the other hand, circRNA has been a novel regulator in the TME, and the interaction between circRNA and CAFs is worth exploring [62,63]. In summary, studying the regulation of exosome secretion of CAFs is beneficial to dissect the tumor–stromal interaction in the TME and support the application of cancer therapy.

CAFs also modify tumor metabolism by secreting specific metabolic substrates including glutamine and its metabolite ammonia [64], alanine [65], aspartate [66], or lysophosphatidylcholine [67]. All of these contribute to different signaling or metabolic pathways, and profoundly affect the biochemical properties of tumor cells.

#### 2.2.3. Angiogenesis

Angiogenesis is a critical process in tumor growth and metastasis, providing adequate nutrients, oxygen, and other metabolic substrates for tumors (Figure 2). This process is mainly initiated by the secretion of vascular endothelial growth factor A (VEGFA) [68]. All of the processes are closely associated with the surrounding tumor stroma.

CAF is an abundant source of VEGFA and other pro-angiogenetic factors such as platelet-derived growth factor C (PDGFC), fibroblast growth factor 2 (FGF-2), and C-X-C motif chemokine 12 (CXCL12/SDF-1), which directly or indirectly regulates tumor neovascularization [2,6,68]. CAF-derived PDGFC, together with VEGFA, mediates tumor growth, metastasis, and angiogenesis [69]. In colon cancer, CAF-derived IL-6 induces VEGFA production in an autocrine or paracrine manner [70], and CAF-derived IL-8 is induced by the autocrine Chitinase 3-like 1 [71], which both promote tumor angiogenesis. CAFs in a secondary site of metastasis promote the process of angiogenesis [72]. Wingless-type MMTV integration site family member 2 (WNT2), a protein that leads to autocrine activation of the Wnt/β-catenin pathway, is upregulated in CAFs and promotes angiogenesis, resulting in promoting colorectal cancer growth in vivo [73]. Taken together, developing strategies against angiogenesis are currently being pursued by oncologic scientists to further shut down cancer-related mortality.

#### 2.2.4. Immunomodulation

Recently, immunotherapy has frequently come into our focus as a cancer treatment. The response rate of immunotherapy is closely associated with the TME. Still, CAFs play the critical role of establishing a pro-tumorigenic niche by modulating multiple immune cells and the ECM to shape an immunosuppressive environment and enhance the resistance to immunotherapy of cancer [74,75]. Tumor-promoting myeloid cells such as M2 tumor-associated macrophages (TAMs), myeloid-derived suppressor cells (MDSCs), and tumor-infiltrating lymphocytes (TILs) are recruited by CAFs. On the other hand, CAFs also abrogate the function of tumor-retarding cytotoxic T cells or NK cells. All of the processes can be mediated by indirect signaling transduction or direct regulation of membrane-bound molecules. For example, CAFs accumulate tumor-promoting immune cells by the activation of multiple pathways such as IL-8 [76], CCL2 [77,78], stromal cell-derived factor (SDF)-1a/CXCR4 [79], and caveolin-1 [80]. CAFs also reprogram the MDSCs with 5-lipoxygenase [81]. Macrophages also reciprocally influence CAF pro-metastasis ability by specific signaling such as Oncostatin M [82]. CAFs’ secrete cytokines or chemokines to interfere with tumor-retarding T cells functions, such as PGE2, indoleamine 2,3-dioxygenase [83], CXCL12 [84], and IL-6 [85]. CAFs also express immune checkpoints such as PD-L1, PD-L2 [86], and B7-H3 [87] to interact with cytotoxic T cells [74]. Intriguingly, a distinct subset of CAFs expresses MHCII molecules to present an antigen to cytotoxic CD4+ T cells; however, this subset of CAFs lacks the costimulatory molecules and restricts the activation of T cells [88].

The above processes finally produce an immunosuppressive and tumor-favorable microenvironment. Recently, studies are increasingly focusing on the distinctive interaction of CAFs and adjacent immune cells, particularly myeloid cells or macrophages. Identification of these mechanisms might provide a new insight for CAF-targeting immunotherapies (Figure 2).

## 3. Targeting CAFs with Natural Compounds

**Figure 3:**
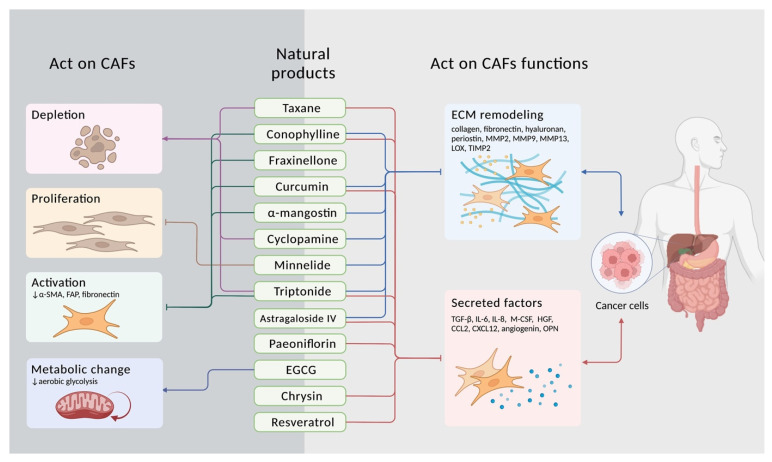
Natural products targeting CAFs to treat digestive system cancer. Several natural products might have therapeutic advantages to digestive system cancer progression by targeting CAFs. Most of the listed natural products have been regarded as health-beneficial compounds. Some of them have been utilized as chemotherapy such as taxane; others have been considered as traditional Chinese medicines (TCM) for a long time, such as triptolide and astragaloside IV; others are combined with chemotherapy as adjuvant therapy, like conophylline, curcumin, cyclopamine, and resveratrol. The antitumor functions of these natural products can be divided into two main aspects: (1) Some natural products act on CAFs depletion, proliferation, activation, or changing CAFs’ metabolism. (2) Some products target CAFs’ functions of ECM remodeling and paracrine secretion. The symbol (↓ in the figure) is representative of the downregulation effect due to the treatment of natural compounds.

Considering the importance of the modulatory effect of the TME and CAFs, therapeutic strategies targeting CAFs are thriving. Current strategies against CAFs include direct depletion, inactivation, and normalization of CAFs [89,90], and those indirectly interfering with the functions of CAFs, such as cytokine production [91]. However, the treatment options are still limited and needed further expansion [92]. Apart from the conventional cancer treatment options, such as chemotherapy or radiotherapy, some studies have turned to focus on natural compounds derived from plants and traditional Chinese medicine (TCM) [93]. Natural compounds have long been considered a rich and cost-effective source of therapeutic compounds, which exert both efficacy and safety [94]. Studies showed that over one-third of the recent anti-cancer drugs are of natural origin [92]. Natural compounds have distinct properties different from synthetic molecules. For example, the high rigidity increases the ability to target protein–protein interactions. The versatile structural complexity and diversity also might be suitable for participating in the biological process and enhancing the therapeutic activity [95,96]. These natural products usually influence multiple signaling pathways and functions in the TME, playing a comprehensive role in suppressing cancer progression [9]. In addition, studies demonstrated that the combination of natural products and chemotherapy drugs reduces toxicity and ameliorates drug resistance [94,97]. Recently, natural products have also displayed a wide range of activities to remodel the TME [93]. Here, we demonstrate several kinds of promising natural compounds, with or without combining other chemotherapy drugs, affecting CAF functions of digestive system cancers. (Figure 3 and Table 1).

### 3.1. Taxane

The taxane family of diterpenoids, including paclitaxel (PTX), docetaxel (DTX), and cabazitaxel, is isolated from the *Taxus* spp. (the Yews) and is known as an effective antitumor substance for the interrupting effect of tubulin polymerization during cell mitosis [117].

The most well-known taxane is PTX extracted from the *Taxus brevifolia* (the Pacific yew), which is used as a chemotherapy drug for ovarian, breast, lung, and other cancers [118]. Due to the severe adverse reactions of the direct infusion of PTX, the nanoformulation of albumin-PTX (also called nab-PTX) has been used for a higher response rate and patient tolerability [119,120]. PTX is applied with other chemotherapy medications, such as gemcitabine, because of the higher survival rates in pancreatic ductal carcinoma and other desmoplastic cancers [121]. Recently, PTX has been shown to suppress the expression of α-SMA and collagen I synthesis by modulating TGF-β/Smad signaling in liver fibrosis [122] and tumor desmoplasia [123]. The findings highlight the potential of PTX in regulating CAFs in the tumor stroma. Clinical data have shown that nab-PTX plus gemcitabine improved the overall survival and response rates compared with gemcitabine alone in pancreatic ductal carcinoma patients [124]. A retrospective study of 65 patients showed that nab-PTX combined with a gemcitabine regimen significantly decreased the density of α-SMA+ fibroblasts and tumor activity [98]. The study indicates the modulatory effect of nab-PTX on CAFs. In addition, nab-PTX targets the cancer–stromal interaction and attenuates the tumor-promoting functions of CAFs. A study using an in vitro co-cultured system demonstrated that nab-PTX inhibited CAF-induced cancer cell migration, invasion, and EMT phenotype by increasing the expression of CXCL10 in cancer cells to counteract the effect of IL-6 secreted by CAFs [99].

DTX belongs to the taxane family and is also clinically approved in cancer chemotherapy. A nanoparticle form of DTX has also been found to target stromal cells and deplete SMA+ fibroblasts and macrophages in pancreatic cancer stroma [100].

### 3.2. Conophylline

Conophylline (CnP) is isolated from the leaves of the tropical plant *Ervatamia microphylla* and belongs to a subset of vinca alkaloids, which is also a kind of chemotherapy drug. The anticancer effect of vinca alkaloid is mainly through inhibiting the polymerization of tubulin and interfering with the cell proliferation process. CnP has been demonstrated to have an antifibrotic effect in liver and pancreatic cancer; therefore, CnP becomes the target of CAF-depleting therapy [125]. In hepatocellular carcinoma, CnP inhibits the expression of α-SMA, the activation marker of CAFs, and the tumor-promoting cytokine production such as IL-6, IL-8, and CCL-2 of CAFs [101]. A similar effect has also been described in the in vitro and in vivo models of pancreatic cancer. The results indicated that CnP suppresses the expression of α-SMA and collagen I synthesis in CAFs and hampers the cancer–stromal crosstalk by decreasing the secretion of IL-6, IL-8, CCL2, and CXCL12. Additionally, CnP combined with gemcitabine inhibited tumor growth and the desmoplasia process, which shows the potential of developing CnP as an adjuvant antitumor drug [102].

### 3.3. Fraxinellone

Fraxinellone (FRA), which is isolated from the root bark of the plant Dictamnus dasycarpus, is a member of the limonoids family. The health benefits of neuroprotective, antifibrotic, anti-inflammatory, and antitumor functions have been demonstrated in several studies. It has been reported that FRA suppresses cancer cell proliferation and angiogenesis and inhibits the immune checkpoint PDL1 expression in several cancer models [126]. Therefore, FRA has the potential to be developed as an effective antitumor drug.

FRA regulates TGF-β signaling in liver fibrosis [127], which indicates the possibility of applying FRA in treating CAF-induced desmoplasia. It has recently been found that a kind of FRA-loaded nanoparticle inactivates CAFs by inhibiting TGF-β signaling in pancreatic ductal carcinoma. Other effects conclude tumor growth inhibition, enhancement of perfusion and drug penetration, and prolonging the survival in a mouse model in the TME [103].

### 3.4. Curcumin

Curcumin is a yellow polyphenol compound refined from the rhizomes of spice *Curcuma longa* (Turmeric) of the ginger family and has long been considered a health-benefitting spice. The biological effects of curcumin include antioxidant, anti-inflammatory, and neuroprotective qualities, which are used in treating metabolic syndrome and obesity. The most outstanding function of curcumin is its anticancer effects via targeting multiple molecular signaling pathways and cell-cycle-related proteins [128]. Several clinical trials are still ongoing to reveal the advantages of curcumin as a complementary therapy of current cancer therapies to improve the efficacy and reduce the side effects [129]. For example, a randomized phase II trial demonstrated the therapeutic potential of curcumin in combination with FOLFOX chemotherapy in metastatic colorectal cancer [130]. Different curcumin nanoparticles are evolving to solve the limitation of low bioavailability and poor water solubility of curcumin [131]. Curcumin has been used as adjuvant therapy of gemcitabine in advanced pancreatic cancer patients and improves the efficacy of traditional chemotherapy [132]. Recent evidence shows other anticancer mechanisms of curcumin, such as immunosuppression [133] and regulation of the EMT via TGF-β-dependent or -independent pathways [134]. We are interested in whether curcumin also regulates the TME of CAFs. It has been reported that curcumin inhibits TGF-β/Smad 2 signaling, and consequently results in multiple anti-invasive effects, such as inhibiting NF-κB and the EMT markers’ expression, decreasing stemness, and promoting 5-FU chemosensitization in colorectal cancer cells co-cultured systems [105]. In addition, curcumin plus doxorubicin in a liposomal formulation also suppresses colon cancer cell proliferation by inhibiting transcriptional factors NF-κB and AP-1 [135]. In the pancreatic cancer model, curcumin downregulates the expression of α-SMA and vimentin, and interferes with the secretion function of CAFs, which results in suppression of the EMT and metastatic phenotype of cancer cells [104]. Both studies indicate that curcumin might have therapeutic potential for hampering the crosstalk between cancer cells and CAFs.

### 3.5. Mangostin

Mangostin is a xanthone derived from a common tropical fruit Mangosteen (*Garcinia mangostana*). The major and most-studied form of mangostin is α-mangostin (α-MG), which has been described to have antibacterial, antifungal, anti-inflammatory, antioxidant, antiobesity, cardioprotective, and anticancer effects [136]. Studies have demonstrated that the antitumor mechanisms of α-MG include apoptosis induction, angiogenesis inhibition, ECM modification, epithelial–mesenchymal transition, and so on [137]. One study has pointed out the effect of α-MG in remodeling tumor stroma recently. A nanoformulated α-MG inhibits TGF-β/Smad signaling and promotes drug-delivery efficacy and vascular perfusion through the CAF inactivation and ECM reduction in pancreatic cancer [106].

### 3.6. Cyclopamine

Cyclopamine is a steroid alkaloid derived from *Veratrum californicum* (false hellebore) and other natural plants. As the first small-molecule inhibitor discovered from the Hh-signaling pathway [138], cyclopamine has been considered a promising therapeutic agent of specific Hh-overexpressing cancers, such as cholangiocarcinoma, osteosarcoma, pancreatic, breast, and colon cancers. In addition, Hh signaling plays a critical role in CAFs’ proliferation and the tumor-promoting functions indicated the potential of cyclopamine to remodel tumor parenchymal tumor stroma as well [139,140]. Co-delivered cyclopamine and PTX nanoparticles in pancreatic cancer suppress cancer growth and modulate tumor stroma by disrupting cancer–stroma crosstalk, decreasing ECM stiffness, and improving blood perfusion, thus increasing the survival rate [107].

### 3.7. Triptolide, Minnelide, and Triptonide

Triptolide, triptonide, and minnelide are extracted from *Tripterygium wilfordii* (thunder god vine), which belongs to a subset of diterpenoids and has been used as a TCM herb to treat several inflammatory and autoimmune diseases for a long time [141].

Triptolide expresses immunosuppressive and antileukemic activity, as well as a most prominent anticancer effect due to the inhibition of transcriptional factors and specific signaling pathways [141,142]. Because of the low solubility of triptolide, a water-soluble prodrug, minnelide is used in the treatment of pancreatic and liver cancer, and several clinical trials are currently ongoing [143]. Minnelide regulates gene expression and distinct signaling pathways in the tumor stroma. For example, triptolide or minnelide inhibits the DNA super-enhancer (SE) in tumor cells and CAFs, which results in downregulation of the critical proto-oncogene MYC and other SE-related genes in pancreatic ductal carcinoma [108]. Minnelide alters CAFs’ activation and proliferation by inhibiting the TGF-β and retinoic acid receptors/retinoid X receptors’ pathways and induces tumor apoptosis by inhibiting the NF-κB pathway [109]. In addition, minnelide reduces the amount of ECM components hyaluronan and collagen and increases the perfusion and drug delivery in pancreatic cancer [110]. A bioinformatics study indicated the potential of triptolide remodeling TME by targeting CXCR4 and cancer-related p53 signaling in gastric adenocarcinoma [144]. Triptonide is another derivative of the thunder god vine. Triptonide treatment was reported to inhibit gastric cancer colony formation, migration, and invasion properties by suppressing the gastric CAF secretion function and regulating miRNA expression [111].

### 3.8. Astragaloside IV

Astragaloside IV is a steroidal triterpene saponin [145], which is the main active compound isolated from *Astragalus membranaceus* (Mongolian milkvetch) and has been mainly used as a TCM herb [146]. The pharmacological effects of astragaloside include antiasthma, anti-inflammatory, cardioprotective, neuroprotective, and antioxidant effects. Astragaloside IV has been demonstrated to counteract TGF-β-induced EMT in mesothelial [147] and gastric cancer cells [148]. Astragaloside IV also suppresses the EMT process in colorectal cancer by modifying micro-RNA activity [149]. The regulating effect of astragaloside IV in the TME has been reported in gastric cancer. Astragaloside IV reestablishes the activity of micro-RNA expression to attenuate the tumor-promoting and tumor secretion functions of CAFs in gastric cancer [112].

### 3.9. Paeoniflorin

Paeoniflorin is a pinane monoterpene glycoside derived from the plants of the *Paeoniaceae* family [150]. Paeoniflorin has been demonstrated to have anti-inflammatory, antioxidative, antiplatelet, and antitumor activities. For example, paeoniflorin inhibits cancer cell survival and invasion by downregulation of the S-phase kinase-associated protein 2 in liver cancer cells [151], and by targeting the Hippo pathway in gastric cancer [152]. Paeoniflorin is reported to interfere with the pro-metastasis functions of CAFs in gastric cancer stroma by the inhibition of IL-6 and micro-RNA-149 [113].

### 3.10. Epigallocatechin-3-Gallate

Epigallocatechin-3-gallate (EGCG) is major catechin extracted from green tea leaves. The antioxidant is the most well-known health benefit effect of catechin. Moreover, EGCG is described to inhibit the effects of several cancers, especially in interfering with aerobic glycolysis activity [153]. For example, EGCG inhibits the survival of gastric cancer and esophageal cancer cells [154,155], inhibits the metabolism of hepatocellular cancer cells, and promotes apoptosis [156].

Recently studies have shown that EGCG suppresses the aerobic glycolytic process in CAFs by inhibiting enzyme phosphofructokinase as well as decreasing the tumor-promoting activity in CAFs, resulting in hampering the proliferation and migration activity of colorectal cancer cells [114].

### 3.11. Chrysin

Chrysin is found in several plants and in honey and classified as a member of the flavonoids, which is described to exert multiple biological effects such as anti-inflammatory, antioxidant, antidiabetic, hepatoprotective, and anticancer activity [157]. Chrysin induced cancer cell apoptosis in colorectal cancer and gastric cancer [158,159]. In hepatocellular carcinoma, one of the synthetic analogs of chrysin named 8-bromo-7-methoxychrysin suppressed the activation of hepatic stellate cells to CAFs and decreased the level of stemness of cancer cells by modifying IL-6 and HGF signaling [115].

### 3.12. Resveratrol

Resveratrol is a stilbenoid polyphenol that was first refined from the rhizomes of *Veratrum grandiflorum* (white hellebore) and has also been found in other plants such as peanuts, grapes, berries, several foods, and red wine. Resveratrol is proposed to have multiple bioactive activities, such as anti-inflammatory, antibacterial, antifungal, and antioxidant effects, and is associated with therapies for neurological disorders, cardiovascular diseases, metabolic syndrome, and cancers [160]. Research shows that resveratrol targets multiple pathways related to inflammation and cancer progression, and several clinical trials are continuing [161]. Resveratrol has pleiotropic mechanisms for cancer therapy [162]. Some studies focus on the synergistic effect with other chemotherapies. For example, uracil phosphoribosyltransferase (EndoCD)/5-FC/resveratrol combination therapy depletes the tumor stroma and sensitizes cancer cells to chemotherapy [163]. Another study points out the antifibrosis and anti-inflammatory effects through regulating several signaling pathways such as TNF-α, IL-6, and NF-κB in treating liver disease [164]. Resveratrol was reported to decrease CAF pro-tumorigenic factor IL-6 and inhibit cancer cell EMT and migration in cholangiocarcinoma [116].

Numerous natural products display modulatory effects on CAFs and the TME. Apart from the effect on digestive system cancers, some of them can be applied to other types of cancers. For example, curcumin increased the level of tumor-suppressor proteins and inhibited CAF activation and paracrine functions in breast cancer [165]. Curcumin inhibits CAF viability by inducing ROS stress in the endoplasmic reticulum [166]. Resveratrol also suppressed the secretion of CAFs leading to the inhibition of breast cancer cell migration and invasion [167]. Cyclopamine also served as a smoothened antagonist and attenuated the effect of CAF-induced EMT in non-small-cell lung cancer cells [168]. These results point out the additional potential to study and develop novel cancer therapies in the future.

## 4. Conclusions

The TME, or tumor stroma, has a profound effect during cancer initiation, progression, and invasion. CAFs are probably one of the most abundant compounds in the tumor stroma and display multifaceted and mainly cancer-promoting functions. CAFs are derived from several kinds of cell types activated by specific signaling and can be classified into different subpopulations according to functions and surface markers. Generally, CAFs interact with the surrounding environment, cancer cells, and immune cells via soluble factors and exosomes’ secretion, or via membrane receptors to deliver cellular signaling and physical cell–cell interactions. Therefore, we can categorize the functions of CAFs into four main groups: ECM remodeling, tumor–stroma crosstalk, angiogenesis, and immunomodulation. Most researchers exploring CAF functions focus on colorectal and pancreatic cancers, probably because of the frequency and fatality of these cancers. According to these studies, CAFs are a promising target to recognize the essence of the biological functions of the TME and further develop novel antitumor therapy strategies.

Numerous natural products have been developed as cancer therapies for decades and have specific properties. Besides targeting cancer parenchyma, several natural products have been discovered to have therapeutic effects on the TME. They interfere with CAF activation, differentiation, tumor-promoting functions, and tumor–stroma crosstalk. Most of the natural compounds have multiple regulatory roles in remodeling CAFs’ characteristics or functions, which might elevate the effect of anti-tumor activities. Therefore, natural products might be a favored source of novel anti-cancer drugs. For example, the common chemotherapy drugs, PTX and CnP belonging to vinca alkaloids, have been shown to have modification effects on CAFs. Several phase II clinical trials show that curcumin has potential as a chemotherapy adjuvant, which can enhance the efficacy of the therapy. Other natural compounds and TCMs, such as FRA, triptolide, resveratrol, cyclopamine, and epigallocatechin-3-gallate, also exert different anti-CAF effects. However, the distinct characteristics of natural products pose both advantages and challenges for clinical application. Most of the studies are still experimental, and the availability of the natural compounds is restricted by some technical issues, such as bioavailability, water solubility, and precision. Therefore, some of the studies tried to develop a nanoparticle form of these compounds to ameliorate these limitations. Currently, there are plentiful, but still unknown, aspects of CAFs waiting to be explored, and the anti-CAFs therapies still face challenges due to the high heterogeneity in the TME. However, all of them might be considered a reflection of the extensive potential for unveiling the mechanisms of tumorigenesis and cancer progression, and consequently furnishing the development of novel cancer therapy. In summary, this review briefly summarizes the current findings of CAF biology and the specific natural products targeting CAFs. We hope this article will provide the impetus for the application of future CAF research and their biological functions and facilitate the advancement of effective cancer treatment options.

## Figures and Tables

**Table 1:** Natural products targeting CAFs.

Drug	Cancer Model	Function		Mechanism	Ref.
		Act on CAFs	Act on CAFs Functions		
Taxane	Pancreatic	Depletion	-	-	[98]
	Pancreatic	-	↓IL-6	-	[99]
	Pancreatic	Depletion	-	-	[100]
Conophylline	Liver	inactivation (α-SMA↓)	↓IL6, IL8, CCL2, angiogenin, OPN	↓GPR68	[101]
	Pancreatic	inactivation (α-SMA/collagen I↓)	↓IL6, IL8, CCL2, CXCL12, TGFβ ECM (↓collagen I)	-	[102]
Fraxinellone	Pancreatic	Inactivation (αSMA/FAP/fibronectin↓)	-	↓TGF-β pathway	[103]
Curcumin	Pancreatic	inactivation (α-SMA/VIM↓)	-	-	[104]
	Colorectal	-	↓MMP13, TGF-β3	↓NF-κB ↓TGF-β pathway	[105]
α-mangostin	Pancreatic	Inactivation (α-SMA/FAP/fibronectin↓)	ECM (↓fibronectin/collagen)	↓TGF-β pathway	[106]
Cyclopamine	Pancreatic	Depletion	ECM (↓LOX/hyaluronan)	↓Hh pathway	[107]
Triptolide Minnelide	Pancreatic	-	↓SE-related genes ↓SE-related protein (BRD4/RNA pol II/COL1A2)	↓DNA SE	[108]
Minnelide	Pancreatic	inactivation (α-SMA↓) inhibit proliferation	ECM (↓collagen/fibronectin/periostin/hyaluronan/MMP2/MMP9)	↓TGF-β & RAR/RXR pathway	[109]
	Pancreatic	Depletion inactivation (α-SMA↓)	ECM (↓hyaluronan/collagen)	↓HAS	[110]
Triptonide	Gastric	-	ECM (↑TIMP2) ↓IL-6	↓miR-301a ↑miR-149	[111]
Astragaloside IV	Gastric	-	ECM (↑TIMP2) ↓M-CSF	↓miR-301a ↑miR-214	[112]
Paeoniflorin	Gastric	-	↓IL-6	↑miR-149	[113]
EGCG	Colorectal	↓aerobic glycolysis	-	↓PFK	[114]
Chrysin	Liver	-	↓IL-6/HGF	-	[115]
Resveratrol	Bile duct	-	↓IL6	-	[116]

Postscript: The symbol (↓ in table) is indicated as downregulated level due to the treatment with the natural compound. On the contrary, the symbol (↑ in table) is indicated as upregulated level due to the treatment.

## Data Availability

Not applicable.
